# Bronchodilator Response Predicts Longitudinal Improvement in Small Airway Function in World Trade Center Dust Exposed Community Members

**DOI:** 10.3390/ijerph16081421

**Published:** 2019-04-20

**Authors:** Deepak Pradhan, Ning Xu, Joan Reibman, Roberta M. Goldring, Yongzhao Shao, Mengling Liu, Kenneth I. Berger

**Affiliations:** 1 Department of Medicine, Division of Pulmonary, Critical Care and Sleep Medicine, New York University School of Medicine, New York, NY 10016, USA; deepak.pradhan@nyumc.org (D.P.); joan.reibman@nyumc.org (J.R.); roberta.goldring@nyumc.org (R.M.G.); 2 Department of Population Health, New York University School of Medicine, New York, NY 10016, USA; Nina.xu@bucknell.edu (N.X.); yongzhao.shao@nyumc.org (Y.S.); mengling.liu@nyumc.org (M.L.); 3 André Cournand Pulmonary Physiology Laboratory, Bellevue Hospital, New York, NY 10016, USA

**Keywords:** airway physiology, dust, environmental health, forced oscillation, respiratory function, small airway disease

## Abstract

The evolution of lung function, including assessment of small airways, was assessed in individuals enrolled in the World Trade Center Environmental Health Center (WTC-EHC). We hypothesized that a bronchodilator response at initial evaluation shown by spirometry or in small airways, as measured by forced oscillation technique (FOT), would be associated with improvement in large and small airway function over time. Standardized longitudinal assessment included pre and post bronchodilator (BD) spirometry (forced vital capacity, FVC; forced expiratory volume in 1 second, FEV_1_) and FOT (resistance at 5 Hz, R_5_; resistance at 5 minus 20 Hz, R_5–20_). Longitudinal changes were assessed using linear mixed-effects modelling with adjustment for potential confounders (median follow-up 2.86 years; 95% measurements within 4.9 years). Data demonstrated: (1) parallel improvement in airflow and volume measured by spirometry and small airway function (R_5_ and R_5–20_) measured by FOT; (2) the magnitude of longitudinal improvement was tightly linked to the initial BD response; and (3) longitudinal values for small airway function on FOT were similar to residual abnormality observed post BD at initial visit. These findings suggest presence of reversible and irreversible components of small airway injury that are identifiable at initial presentation. These results have implications for treatment of isolated small airway abnormalities that can be identified by non-invasive effort independent FOT particularly in symptomatic individuals with normal spirometry indices. This study underscores the need to study small airway function to understand physiologic changes over time following environmental and occupational lung injury.

## 1. Introduction

The WTC Environmental Health Center (WTC-EHC) is a program for treatment of community members with medical and mental health symptoms as a result of exposure to the disaster that occurred in New York on 9/11/2001. Lower respiratory symptoms (LRS) have been reported in WTC dust exposed community members and responders [1,2,3,4,5,6,7,8,9,10]. There are multiple reports of changes in spirometry associated with LRS, and importantly, small airway abnormalities have been associated with LRS even in the presence of normal spirometry [4,5,6,8,11,12,13]. Longitudinal improvement in LRS and spirometry is reported in many, although symptoms have often persisted [3,5,14,15]. Of note, structural changes within the airways and/or parenchyma have been demonstrated in community members, suggesting a potentially irreversible component of lung injury [16]. These findings suggest an admixture of reversible and irreversible components of lung injury in response to WTC dust exposure. 

We hypothesized that a bronchodilator response at initial evaluation shown by spirometry or in small airways, as measured by forced oscillation technique (FOT), would be associated with improvement in large and small airway function over time. FOT was selected as a noninvasive test based on demonstrated ability to capture small airway dysfunction in WTC dust exposed populations even when airflow remains normal when assessed by spirometry [4,5,11,17,18]. Reduction of airflow on spirometry may not occur in the setting of isolated small airway disease due to the large aggregate cross-sectional area of the distal airways [19,20,21]. FOT overcomes this limitation by providing measures of non-uniformity of airflow distribution that reflect regional functional abnormalities in the small airways [22,23,24].

Based on the above considerations, longitudinal data for lung function using spirometry and FOT to capture small airway function were analyzed in community members enrolled in the WTC EHC. The study was designed to determine: (1) whether FOT measures of small airway function improved over time, and (2) whether the bronchodilator response at initial evaluation was associated with a reversible component of lung injury shown by longitudinal improvement in lung function.

## 2. Materials and Methods

### 2.1. Patients

Adult patients were self-referred to the WTC EHC with medical and/or mental health symptoms related to September 11, 2001 exposures as previously described [2]. Patients were included in this analysis if they had valid spirometry and FOT, which included bronchodilator testing at their initial evaluation and at least one subsequent spirometry and FOT evaluation. The Institutional Review Board of New York University School of Medicine approved the research database (NCT00404898), and only data from patients who provided informed consent were used for analysis. 

### 2.2. Procedures

At initial visit to the WTC EHC patients completed a multi-dimensional interviewer-administered questionnaire that included questions relating to demographic characteristics, WTC-related dust exposures, presence and severity of respiratory symptoms, and history of tobacco use [2]. Patients were treated for asthma-like symptoms according to guidelines for asthma management [25], and evaluated with further additional studies if findings were inconsistent with asthma.

### 2.3. Spirometry and FOT Measurements

At initial visit all patients were referred for objective assessment of lung function using spirometry and FOT which was performed within a single location (André Cournand Pulmonary Physiology Laboratory, Bellevue Hospital). Spirometry and FOT were routinely performed on all patients before and 15 min after bronchodilator (BD) administration (2.5 mg albuterol sulfate delivered via nebulizer over 5 min) according to standard ATS/ERS guidelines [26,27]. FOT is a noninvasive test that measures the relationship between pressure and airflow fluctuations applied externally to the respiratory system during tidal breathing to determine the respiratory system resistance. Measurements included resistance assessed at an oscillating frequency of 5 Hz (R_5_) and frequency dependence of resistance calculated as the difference between resistance at 5 and 20 Hz (R_5–20_). R_5–20_ provides a measure of non-uniformity of airflow distribution that correlates with frequency dependence of compliance measured by invasive esophageal manometry, an established test of small airway function [22,23,24,28,29].

Predicted values for spirometry measures were derived from NHANES III [30]. and abnormal spirometry was defined by forced expiratory volume in 1 second (FEV_1_), forced vital capacity (FVC), or FEV_1_/FVC measurements below the lower limit of normal (LLN) [31]. A positive BD response (+BD) on spirometry was defined according to ATS/ERS guidelines as a ≥12% and ≥200 cc increase in FEV_1_ and/or FVC after bronchodilator [26]. FOT was performed under tidal breathing (Jaeger IOS^®^, Vyaire Medical, Yorba Linda, CA, USA). Measurements included respiratory resistance at 5 Hz (R_5_) and the difference in resistance from 5 to 20 Hz (R_5–20_) as an index of frequency dependence of resistance (FDR). Upper limits of normal (ULN) for R_5_ and R_5–20_ (0.396 kPa/L/s and 0.075 kPa/L/s, respectively) were based on measures in asymptomatic non-smoking subjects with normal spirometry in our laboratory (*n* = 80) and the values are in the range of published estimates of normative data [18,32]. A positive BD response for IOS measurements was defined as a decrease in R_5_ of ≥0.135 kPa/L/s, based on the 95th percentile for BD response in healthy adults in normative data on forced oscillation [32].

### 2.4. Definitions

WTC dust cloud exposure was defined by patient report of having been caught in a dust cloud created by a building collapse on 9/11. Patients were further classified as local residents, local workers or clean-up workers based on their description of location and activities. New-onset lower respiratory symptoms were defined by the presence of at least one symptom of wheezing, chest tightness, or dyspnea with onset after 11 September 2001. Symptomatic patients at initial presentation to the WTC EHC were defined based on a symptom frequency ≥2 times per week in the four preceding weeks. Patients were classified as a smoker if they had >5 pack year lifetime history of tobacco use.

### 2.5. Statistical Methods

Continuous variables were summarized using median and interquartile range (IQR) and compared across groups using nonparametric Kruskal-Wallis test. Categorical variables were summarized by counts and proportions and compared using Chi-squared test. A linear mixed-effects model was used to investigate longitudinal changes in lung function using each repeated spirometry and FOT measurement. In each model a fixed linear effect of the follow-up time (defined as duration since initial visit) on a measure of respiratory function was estimated with adjustment for potential confounders. Among the considered confounders, gender, race/ethnicity, income, WTC exposure category, dust cloud exposure did not substantively change the results and thus only age, BMI, and smoking history were included in the final models. Random intercept and slope were assumed to explain within-subject correlation among repeated measurements and among-subject heterogeneity.

Separate models were fit with pre-BD FVC, FVC% of predicted, FEV_1_, FEV_1_% of predicted, and FEV_1_/FVC as dependent variables. To analyze longitudinal changes in FOT parameters, similar models were fit with pre-BD R_5_ and R_5–20_ as the dependent variables. A *p* value < 0.05 was used to indicate statistical significance. Analyses were conducted using R (version 3.0.2, R Foundation for Statistical Computing, Vienna, Austria) and SPSS^®^ (version 20.0, IBM^®^ Corp, Armonk, NY, USA).

## 3. Results

### 3.1. Patient Demographics and Characteristics

A total of 1146 adult patients were enrolled in the WTC EHC between 29 October 2002 and 20 February 2013 who fit inclusion criteria. Patients were subsequently excluded from analysis if their studies were repeated within 180 days (*n* = 133), if post BD data were not available (*n* = 119), or if longitudinal data for either spirometry or FOT were not available (*n* = 153). The final analytic cohort consisted of 741 adult patients who met criteria for longitudinal study. Patient characteristics are shown in Table 1. The cohort had a median age of 51 at enrollment, with an equal distribution of males and females. Many patients were overweight with a median BMI of 28.1 (IQR 24.7–32.4) kg/m^2^. The cohort was racially and ethnicity diverse, with the largest single group identifying themselves as Hispanic (36%). Less than a quarter (24%) of patients had a smoking history, with few (13%) reporting current tobacco use. The cohort included predominantly local workers (51%), with fewer local residents (20%) and clean-up workers (17.0%). Over half (52%) were caught in the dust cloud caused by the collapse of the World Trade Center towers on 9/11. The group was highly symptomatic with 93% reporting LRS that began after 9/11 and persisted at the time of enrollment in the WTC EHC.

### 3.2. Lung Function at Initial Visit

The results of lung function evaluation at the initial visit are shown in Table 2. Median values for spirometry were within the normal range for pre-BD FVC, FEV_1_, and FEV_1_/FVC (median 92% predicted, 88% predicted, and 77% respectively). The percentage of individuals with values below the LLN for FVC, FEV_1_, and FEV_1_/FVC were 22, 28, and 20%, respectively. Only 58 patients (8%) met ATS/ERS criteria for a positive BD response on spirometry. Since 93% of the subjects developed respiratory symptoms after the WTC attack, data prior to the disaster were generally not available.

In contrast to spirometry measurements, the majority of patients had abnormal oscillometry measurements, with an elevated median pre-BD R_5_ and R_5–20_ (0.489 and 0.098 cm H_2_O/L/sec, respectively), consistent with presence of airway dysfunction not evident on spirometry. The median decrease in these parameters after BD for the total cohort was 14% for R_5_ and 23% for R_5–20_. A positive BD response on oscillometry was noted 163 patients (22% of the cohort). 

### 3.3. Longitudinal Spirometry Measurements

Patients were followed for a median of 2.86 years (IQR 1.8–3.7 years), with 95% of the repeated measurements occurring within 4.9 years. Table 3 shows the results from linear mixed-effects modeling adjusted for age, smoking, BMI, baseline pre-BD FEV_1_ %predicted, and baseline pre-BD FVC %predicted. Spirometry measurements in the cohort improved over time for FVC and FEV_1_ (36 ± 6 mL and 22 ± 4 mL/year respectively, *p* < 0.001); these changes in absolute values corresponded to an improvement in % of predicted FVC = 0.80 ± 0.17% per year and % of predicted FEV_1_ = 0.60 ± 0.17% per year (*p* < 0.001 for both analyses). Subgroup analysis demonstrated that individuals with abnormal spirometry at initial visit had a significantly greater improvement in both FVC and FEV_1_ over time than those with normal spirometry at initial visit (*p* < 0.003 for both variables, data not shown). 

We evaluated whether patients with a positive BD response on spirometry at baseline had greater improvement in spirometry parameters over time compared with those who had a negative BD response. To conduct this analysis, data analyzed were limited to the 270 individuals with abnormal spirometry testing at baseline. Demographic characteristics did not differ between the group with (−) or (+) BD response at baseline. Initial values for FEV_1_ and FEV_1_/FVC were lower in patients with a (+) BD response compared with those with a (−) BD response (FEV_1_ 67% predicted [IQR 54–75] vs 74% predicted [IQR 67–82], *p* < 0.001; FEV_1_/FVC 64% [IQR 59–71] vs 72% [IQR 67–81], *p* < 0.001). Whereas both groups showed improvement in FVC and FEV_1_ over time, the improvement was greater in the individuals with a (+) BD response at baseline compared with those with a (−) BD response (ΔFVC = 122 ± 28 vs. 28 ± 9 mL/year; ΔFEV_1_ 91 ± 7 vs. 16 ± 7 mL/year). Neither group demonstrated longitudinal change in FEV1/FVC. Although robust improvement in spirometry values were noted in the patients with (+) BD response, 70.7% demonstrated longitudinal values for FVC and/or FEV_1_ that remained in the abnormal range. 

### 3.4. Longitudinal FOT Measurements

Table 4 shows results for longitudinal assessment of lung function using oscillometry. In contrast to spirometry, the group as a whole failed to demonstrate change in either R_5_ or R_5-20_. Subgroup analysis of individuals who had abnormal oscillometry at initial evaluation demonstrated decline in resistance when assessed as R_5_ (slope = −0.007 kPa/L/sec per year, *p*< 0.05).

**Figure 1:**
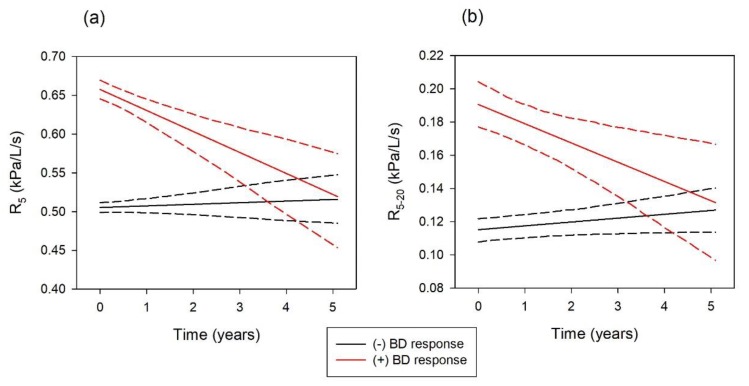
Panel (**a**) Longitudinal values and confidence interval for R_5_ derived from mixed-effects modelling for the (−) BD and (+) BD groups. Panel (**b**) Longitudinal values and confidence interval for R_5–20_ derived from mixed-effects modelling for the (−) BD and (+) BD groups.

The predictive value of a (+) BD response on FOT at baseline was evaluated in the cohort of 527 individuals with abnormal oscillometry testing at baseline. Demographic characteristics did not differ between the group with (−) or (+) BD response at baseline. Although oscillometry was abnormal in all of these patients, more abnormal pre BD values for R_5_ and R_5–20_ were detected at the initial visit in individuals with a (+) BD response compared with those with a (−) BD response (R_5_: 0.685 [IQR 0.573–0.816] vs 0.509 [IQR 0.441–0.597] kPa/L/s; R_5–20_: 0.210 [IQR 0.123–0.302] vs 0.109 [IQR 0.068–0.165 kPa/L/s]. Table 4 and Figure 1 illustrate that while oscillometry variables remained stable in the group with (−) BD response at baseline, the group with (+) BD response at baseline showed significant longitudinal improvement over time (ΔR_5_ = −0.027 ± 0.006 kPa/L/s/year, *p* < 0.001; ΔR_5–20_ = 0.012 ± 0.004 kPa/L/s/year, *p* = 0.002). Additional analysis demonstrated that the post-BD FOT values in these patients remained stable over time indicating that the residual abnormality noted post bronchodilator at baseline remained unchanged over time despite standard medical therapy.

## 4. Discussion

The present study addressed the evolution of lung function including assessment of small airways in a cohort of community members enrolled in the WTC EHC with new onset lower respiratory symptoms following exposure to WTC dust. Improvement in respiratory function was demonstrated by parallel improvement in airflow and volume measured by spirometry as well as resistance (R_5_) and FDR (R_5–20_) measured by oscillometry. Moreover, the magnitude of improvement that occurred with therapy over time was tightly linked to a BD response at the initial visit. Lastly, the longitudinal values for small airway function observed on oscillometry were similar to the residual abnormality observed post BD at initial visit. These findings suggest the presence of reversible and irreversible components of small airway injury that are identifiable at initial presentation.

Data collected in numerous populations exposed to the WTC disaster demonstrate that in many the predominant site of injury is to the small airways. The most common abnormality noted on spirometry in exposed firefighters, workers at the WTC site, and community members was parallel reduction in both FVC and FEV_1_ (i.e., without change in FEV_1_/FVC) [1,2,4,6,8]. Small airway injury was implicated in these populations based on evidence for air trapping on plethysmography and/or computed tomography [1,6,33,34]. In accord with these findings, recent data show that exposure to inhaled ambient particulate matter at sizes below 2.5 μm and occupational toxins are also associated with small airway dysfunction as evidenced by parallel reduction in FVC and FEV_1_ [35,36,37,38,39,40]. Furthermore, studies in WTC exposed populations using oscillometry demonstrate presence of FDR in accord with non-uniform distribution of airflow within small airways [4,11,41,42]. The presence of FDR is tightly correlated with presence of frequency dependence of compliance detected by esophageal manometry, an established marker of small airway dysfunction [11,28,29,43,44]. The clinical relevance of FDR in WTC dust exposed patients has been highlighted by association with magnitude of dust exposure, presence of wheeze and presence of systemic inflammation as assessed by serum C-reactive protein levels [4,17,18]. 

Longitudinal data from the present study indicate improvement in small airway function assessed by both spirometry and oscillometry. Spirometry assessments demonstrated parallel improvement of FVC and FEV_1_ without change in FEV_1_/FVC. This pattern is in accord with prior descriptions of a “volume response” following bronchodilator inhalation in patients with asthma and COPD, which has been attributed to improvement in small airway function with relief of air trapping [45,46,47,48,49]. In the present study, FOT was added to routine assessment of lung function by spirometry to directly assess the role and evolution of small airway function in WTC dust exposed individuals. Longitudinal assessment using FOT demonstrated improvement in FDR, in accord with improved small airway function over time. Of note, this improvement in small airway function was evident only in those individuals who had abnormal respiratory function at baseline with a reversible component, as evident by testing post administration of bronchodilator. 

Prior studies in WTC dust exposed populations have indicated presence of both reversible and irreversible airways injury over time. Registry based longitudinal studies have indicated that resolution of lower respiratory symptoms over time is associated with improvement in measures of small airway function and conversely, persistence of LRS is associated with persistent small airway dysfunction [3,5]. In contrast, persistence of severe LRS has been documented despite a high level of respiratory medication use, including inhaled corticosteroid and long acting β-agonist [3]. Similarly, high rates of poorly or very poorly controlled asthma has been documented in numerous cohorts of individuals that were exposed to WTC dust [50,51,52,53,54]. We have previously demonstrated presence of sustained inflammation in WTC dust exposed community members based on peripheral eosinophilia and increased C-reactive protein levels, findings that were associated with persistent abnormality in small airway function [17,55]. Objective evidence for inhalation of particles to the distal lung and structural airway abnormalities has been demonstrated using analysis of induced sputum and by histologic assessment of lung tissue which showed peribronchiolar fibrosis and emphysematous changes [16,56,57]. Taken together these findings are consistent with a chronic disease syndrome due to disease in the small airways. Lack of complete improvement in small airway function may be due to chronic inflammation that is either not fully corrected by the current medical therapy and/or is located beyond the delivery range of conventional inhaled corticosteroid therapy. Alternatively, given the structural changes in the distal lung noted on histology, the residual small airway dysfunction observed over time could reflect irreversible structural damage that occurred prior to initial evaluation, presumably at time of injury.

There are some factors to consider when interpreting the data in the present study. First, there is significant heterogeneity within this population regarding amount and duration of WTC dust exposures, as well as other potential environmental exposures. However, data remained significant after adjustment for exposure categories, dust cloud exposure and smoking. Second, although medical therapy was prescribed by a standardized clinical algorithm, adherence to therapy was not documented. Of note, medications and provider visits were provided free-of-charge, theoretically minimizing financial and access to care issues as barriers to adherence. Despite these limitations, improvements in spirometry and FOT measures of small airway function were documented and were associated with presence of acute reversibility at baseline. Nevertheless, a contribution from non-adherence to medical therapy to the residual abnormality in both spirometry and FOT cannot be excluded. Third, modeling was performed with a linear time trend for changes in spirometry. These improvements would eventually be counterbalanced by normal age-related decline in spirometry, but analysis over a longer follow-up duration would be needed. In contrast, there are no data demonstrating age-related change in oscillometry in adult populations. Fourth, there is potential for bias as all patients were self-referred to the EHC with potential for follow-up bias as only 83% of patients with valid spirometry and oscillometry measurements at initial visit had subsequent valid spirometry and oscillometry measurements. Finally, this study was not designed to examine the association of longitudinal change in lung function with change in lower respiratory symptoms over time. 

An additional limitation of this study relates to lack of interpretation of FOT data with respect to lung volume; change in values for resistance over time would occur if functional residual capacity (FRC) varied at each study visit. Although FRC was not measured in this study, the most likely explanation for variability over time would related to changing body weight. However, significant changes in body weight has not been evident in patients that have returned for routine monitoring within our WTC EHC population.

## 5. Conclusions

Inhalation of WTC dust was associated with small airway abnormalities in a cohort of exposed community members which showed incomplete improvement over time. Positive bronchodilator response in the small airways at presentation identified individuals with significant ability for improvement in lung function over time; however, residual abnormality noted post-BD at baseline identified an irreversible component of small airway injury. These results have implications for the treatment of isolated small airway abnormalities that can be readily identified by non-invasive effort independent FOT particularly in individuals who are symptomatic with normal spirometry indices. Finally, this study also underscores the need to study small airway function to understand physiologic changes over time for this and other environmental and occupational lung injuries.

## Figures and Tables

**Table 1:** Patient characteristics at initial visit (*n* = 741).

Characteristic	Value
Age, median (IQR)	51 (44–59)
Gender; (M/F, %)	50/50
BMI (kg/m^2^), median (IQR)	28 (25–32)
Race/Ethnicity	
Hispanic	268 (36)
White	241 (33)
Black	155 (21)
Asian	52 (7)
Unspecified	25 (3)
Income/year ≤ $15,000	286 (39)
Smoking history	
>5 pack-year	177 (24)
Current smoker	96 (13)
WTC exposure category	
Local worker	378 (51)
Clean up worker	128 (17)
Resident	149 (20)
Rescue/recovery	25 (3)
Unspecified	61 (8)
Caught in WTC dust cloud	382 (52)
Lower respiratory symptoms	691 (93)

Data are presented at *n* (%) unless otherwise noted. IQR: interquartile range; BMI: body mass index; WTC: World Trade Center.

**Table 2:** Lung function at initial visit (*n* = 741).

	Pre-BD	Post-BD
Spirometry		
FVC (%predicted)	92 (81–103)	92 (81–103)
FEV_1_ (%predicted)	88 (76–99)	92 (80–103)
FEV_1_/FVC	77 (71–81)	79 (74–84)
FOT		
R_5_ (kPa/L/s)	0.489 (0.375–0.615)	0.428 (0.328–0.527)
R_5–20_ (kPa/L/s)	0.098 (0.055–0.168)	0.074 (0.041–0.128)

Data are presented at median (IQR). BD: bronchodilatory FOT: forced oscillation technique.

**Table 3:** Longitudinal change in spirometry parameters.

	Whole Cohort	Abnormal Spirometry		
	(*n* = 741)	Total (*n* = 270)	(−) BD Response (*n* = 212)	(+) BD Response (*n* = 58)
ΔFVC (mL/year)	36 ± 6 *	43 ± 10 *	28 ± 9 **	122 ± 28 *
ΔFEV_1_ (mL /year)	22 ± 4 *	29 ± 7 *	16 ± 7 **	91 ± 7 *

* *p* < 0.001; ** *p* < 0.05.

**Table 4:** Longitudinal change in oscillometry parameters.

	Whole Cohort	Abnormal Oscillometry		
	(*n* = 741)	Total (*n* = 527)	(−) BD Response (*n* = 364)	(+) BD Response (*n* = 163)
ΔR_5_ (kPa/L/s/year)	−0.001 ± 0.002	−0.006 ± 0.003 *	0.002 ± 0.003 *	−0.027 ± 0.006 **
ΔR_5–20_ (kPa/L/s/year)	0.001 ± 0.001	−0.001 ± 0.001	0.002 ± 0.001	−0.012 ± 0.004 *

* *p* < 0.001; ** *p* < 0.05.
